# Mismatch Repair-Deficient Crypt Foci in Lynch Syndrome – Molecular Alterations and Association with Clinical Parameters

**DOI:** 10.1371/journal.pone.0121980

**Published:** 2015-03-27

**Authors:** Laura Staffa, Fabian Echterdiek, Nina Nelius, Axel Benner, Wiebke Werft, Bernd Lahrmann, Niels Grabe, Martin Schneider, Mirjam Tariverdian, Magnus von Knebel Doeberitz, Hendrik Bläker, Matthias Kloor

**Affiliations:** 1 Department of Applied Tumour Biology, Institute of Pathology, University Hospital Heidelberg, Im Neuenheimer Feld 224, 69120 Heidelberg, Germany, and Clinical Cooperation Unit Applied Tumour Biology, DKFZ (German Cancer Research Center) Heidelberg, Im Neuenheimer Feld 280, Heidelberg, Germany; 2 Department of Biostatistics, DKFZ (German Cancer Research Center), Im Neuenheimer Feld 280, Heidelberg, Germany; 3 Bioquant, Hamamatsu Tissue Imaging and Analysis (TIGA) Center, Heidelberg, Germany; 4 Department of General, Visceral and Accident Surgery, University Hospital Heidelberg, Heidelberg Germany; 5 University Hospital Charité, Department of General Pathology, Berlin, Germany; University of Navarra, SPAIN

## Abstract

Lynch syndrome is caused by germline mutations of DNA mismatch repair (MMR) genes, most frequently *MLH1* and *MSH2*. Recently, MMR-deficient crypt foci (MMR-DCF) have been identified as a novel lesion which occurs at high frequency in the intestinal mucosa from Lynch syndrome mutation carriers, but very rarely progress to cancer. To shed light on molecular alterations and clinical associations of MMR-DCF, we systematically searched the intestinal mucosa from Lynch syndrome patients for MMR-DCF by immunohistochemistry. The identified lesions were characterised for alterations in microsatellite-bearing genes with proven or suspected role in malignant transformation. We demonstrate that the prevalence of MMR-DCF (mean 0.84 MMR-DCF per 1 cm^2^ mucosa in the colorectum of Lynch syndrome patients) was significantly associated with patients’ age, but not with patients’ gender. No MMR-DCF were detectable in the mucosa of patients with sporadic MSI-H colorectal cancer (n = 12). Microsatellite instability of at least one tested marker was detected in 89% of the MMR-DCF examined, indicating an immediate onset of microsatellite instability after MMR gene inactivation. Coding microsatellite mutations were most frequent in the genes *HT001* (*ASTE1*) with 33%, followed by *AIM2* (17%) and *BAX* (10%). Though MMR deficiency alone appears to be insufficient for malignant transformation, it leads to measurable microsatellite instability even in single MMR-deficient crypts. Our data indicate for the first time that the frequency of MMR-DCF increases with patients’ age. Similar patterns of coding microsatellite instability in MMR-DCF and MMR-deficient cancers suggest that certain combinations of coding microsatellite mutations, including mutations of the *HT001*, *AIM2* and *BAX* gene, may contribute to the progression of MMR-deficient lesions into MMR-deficient cancers.

## Introduction

Lynch syndrome, occurring with an estimated allele frequency of up to 1:350, is one of the most common inherited cancer syndromes [1] and clinically often referred to as hereditary non-polyposis colorectal cancer (HNPCC) [2]. Lynch syndrome mutation carriers have a lifetime risk of about 40% to 80% to develop colorectal cancer [3,4].

Tumorigenesis in Lynch syndrome-associated malignancies is caused by deficiency of one of the DNA MMR genes, such as *MLH1*, *MSH2*, *PMS2* and *MSH6* [5]. Carriers of a germline mutation in one allele of the respective MMR gene acquire a second somatic mutation that inactivates the remaining functional allele of the MMR gene, following the classical two-hit hypothesis [6]. Inactivation of MMR genes consequently leads to the loss of MMR protein expression in tumour cells and to an MSI-H phenotype. This phenomenon can be detected by immunohistochemistry [7,8] and thus point to the appropriate MMR gene for mutation analysis.

A feature characteristic of Lynch syndrome is the lack of polyposis, which is in sharp contrast to other inherited colorectal cancer-predisposing syndromes like the familial adenomatous polyposis [9–11]. Recently, MMR-DCF have been described as a non-polypous morphologic correlate of somatic MMR gene inactivation in Lynch syndrome [12]. MMR-DCF occur at thousands in otherwise phenotypically normal intestinal mucosa of Lynch syndrome mutation carriers [12]. This is in contrast to the limited penetrance of Lynch syndrome and indicates that MMR-DCF only rarely progress to cancer. However, the natural history of MMR-DCF, as well as their association with clinical parameters and their molecular characteristics, are completely unknown.

At advanced stages of tumour progression, MMR deficiency can promote tumour development through mutations affecting microsatellites located in gene-encoding regions that are essential for growth regulation, differentiation or apoptosis [13–15]. Certain coding microsatellite-bearing target genes have been proven or suspected to have functional relevance in MSI-H colorectal cancer. These include *ACVR2* (*activin receptor type 2*), *TAF1B (TATA box binding protein-associated factor)* and *HT001*, also known as *ASTE1* (*asteroid homolog 1*), which show mutation rates of more than 80% in MSI-H colorectal cancer [15–18]. Also *AIM2* (*absent in melanoma 2*), and the pro-apoptotic *BAX* (*BCL2-associated X protein*) are inactivated by coding microsatellite mutations in the majority of MSI-H colorectal cancer [19–21]. The most prominent example of a functionally relevant target gene in MSI-H colorectal cancer, is the *TGFBR2* (*transforming growth factor receptor type 2*) gene [22] which bears a coding microsatellite of ten adenosine residues frequently targeted by MMR gene inactivation.

In the present study, we aimed to characterise the clinical and molecular characteristics of MMR-DCF as possible cancer precursor lesions in Lynch syndrome. To that end, we correlated the occurrence of MMR-DCF with clinical parameters like patients’ age, gender, and MMR gene germline mutation. In order to identify the consequences of MMR deficiency with potential functional significance in MMR-DCF, we focused on potential driver mutations in selected coding microsatellite-bearing target genes.

## Materials and Methods

### Samples of patient cohorts and controls

Paraffin-embedded archival tissues from small and large bowel (270 paraffin blocks) from Lynch syndrome patients (n = 34) operated for colorectal cancer were retrieved from the archive of the Institute of Pathology University Hospital Heidelberg, Heidelberg, Germany. Resections had been performed between 1999 and 2011. Non-tumorous mucosa from the resection margins and from tumour-adjacent non-neoplastic tissue was analysed for changes in MMR protein expression (MLH1, MSH2, and MSH6). Of the 34 Lynch syndrome patients, 19 showed a *MLH1* mutation, 14 a *MSH2* mutation and one patient presented with a *MSH6* mutation. In addition non-tumorous mucosa was analysed from patients with sporadic MMR-deficient cancers caused by *MLH1* promoter methylation (n = 12). Moreover, control staining with an antibody specific for an MMR protein not affected by a germline mutation was performed in 5 of the Lynch syndrome patients and one MSS control patient. For correlation analysis with clinical parameters, the cohort was enlarged by including 10 patients (LS 1–10; 4 with *MLH1* and 6 with a *MSH2* mutation) and 9 controls (patients with MMR-proficient colorectal cancer) published in our previous study [12]. The study was approved by the Ethics Committee of the University Hospital Heidelberg. All patients provided informed and written consent for participation in the study of the German HNPCC Consortium.

All blocks were cut into ten serial sections of 2 μm each. The fifth slide was chosen for MMR protein immunohistochemistry, three adjacent slides were used for manual microdissection and four slides were stained with antibodies against immune cell markers. These slides together with the fifth one were scanned using the Nanozoomer 2.0-HT Scansystem at the Hamamatsu TIGA Center (BioQuant, Heidelberg) for analysis and later also used for microdissection.

### Immunohistochemistry and length measurement

Immunohistochemistry was performed on 2 μm paraffin sections by using monoclonal antibodies specific for MLH1 (clone G168-15, dilution 1:25, BD Pharmingen, Heidelberg, Germany), MSH2 (clone FE11, dilution 1:200, Calbiochem, Darmstadt, Germany) or MSH6 (clone44, dilution 1:50, Cell Marque, Rocklin, USA) for detecting loss of MMR protein as described previously [12]. An immunoperoxidase method was used to visualise the antibodies by labelling them with a chromogen (3-amino-9-ethylcarbazole, Dako, Glostrup, Denmark). The amount of mucosal length and surface analysed was calculated as described previously [12].

For immunohistochemical staining of immune cells in the vicinity of MMR-DCF the following antibodies were used: CD4 (clone RPA-T4, dilution 1:50, BD Pharmingen, Heidelberg, Germany), CD8 (clone 4B11, dilution 1:50, Novocastra, Wetzlar, Germany) and FoxP3 (clone 236A/E7, dilution 1:50, eBioscience, Frankfurt, Germany).

Immune cell infiltration was qualitatively assessed by two independent observers.

### PCR and direct microdissection

Nine microsatellite markers were examined in MMR-DCF and normal MMR-proficient intestinal crypts by PCR using Platinum Taq Polymerase (Invitrogen, Life technologies, Darmstadt, Germany). For each PCR, the master mix was prepared as follows: 2.5 μl of 10 x buffer, 0.75 μl of 50 mM MgCl_2_, 5 μl of 1.25 mM dNTPs, 0.2 μl of Taq polymerase, two or three primer pairs in different dilutions (see below) and RNAse-free water ad 25 μl. Template was added to the PCR mastermix by direct manual microdissection from immunohistochemically or haematoxylin/eosin-stained sections of MMR-DCF and control crypts.

To increase the number of evaluated genes bearing coding or non-coding microsatellites, different duplex or triplex PCRs were performed using the following primer combinations: Approach 1: AIM2 (1 μl per primer), ACVR2 (3 μl); Approach 2: BAX (1 μl), HT001 (2 μl); Approach 3: TGFBR2 (1 μl), TAF1B (1 μl); Approach 4: BAT25 (0.7 μl), BAT26 (1 μl) and CAT25 (1.3 μl). For approaches 1–3 cycling conditions were as follows: 5 min at 94°C; 40 cycles: 52 s at 94°C, 66 s at 56°C, 44 s at 72°C; 6 min at 72°C. For approach 4, cycling conditions were identical except for the annealing temperature (55°C). Coding microsatellites located within a gene are referred to by the name of the gene throughout the manuscript.

PCR products were separated on an ABI3100 sequencer (Applied Biosystems, Darmstadt, Germany), and fragment lengths were identified with the GeneMapper software 5 (Release 5.0, Applied Biosystems, Darmstadt, Germany). As a control for the molecular analyses, an additional set of 7 MSS colorectal cancer patients was included in the microdissection approach

### Statistical analysis

Since the surface area and the crypt numbers were estimated as functions of mucosal length, the statistical analysis of the number of MMR-DCF was performed with respect to the assessed mucosal lengths only. Based on the assumption that the number of MMR-DCF follows a Poisson distribution, a Poisson regression model was applied to analyse the occurrence of crypt foci with respect to mucosal length, tumour localisation, germline mutation, age and gender. Models were checked for overdispersion and zero inflation. Expected counts and incident rate ratios together with their corresponding 95% confidence intervals (95% CIs) were computed to illustrate the results. Fisher’s exact test was used to compare mutation frequencies of the analysed microsatellite markers in MMR-DCF and controls. For all statistical tests undertaken, a result was regarded significant with a p-value <0.05.

## Results

### Frequency and clinical distribution

In total, MMR protein immunohistochemistry was performed in 714 cm of mucosa (626 cm large bowel, 88 cm small bowel) from carriers of a Lynch syndrome mutation and 144 cm (129 cm large bowel, 15 cm small bowel) from patients without a germline mutation in the respective MMR gene, including 62 cm of mucosa (49 cm large bowel, 13 cm small bowel) from patients with sporadic MSI-H colorectal cancer. This corresponds to the following surface areas: 24.9 cm^2^ mucosa from Lynch syndrome mutation carriers (22.5 cm^2^ large bowel, 2.4 cm^2^ small bowel) and 5.1 cm^2^ from patients without an MMR gene germline mutation (4.7 cm^2^ large bowel, 0.4 cm^2^ small bowel), including 2.2 cm^2^ from sporadic MSI-H colorectal cancer patients (1.8 cm^2^ large bowel, 0.4 cm^2^ small bowel).

**Fig 1:**
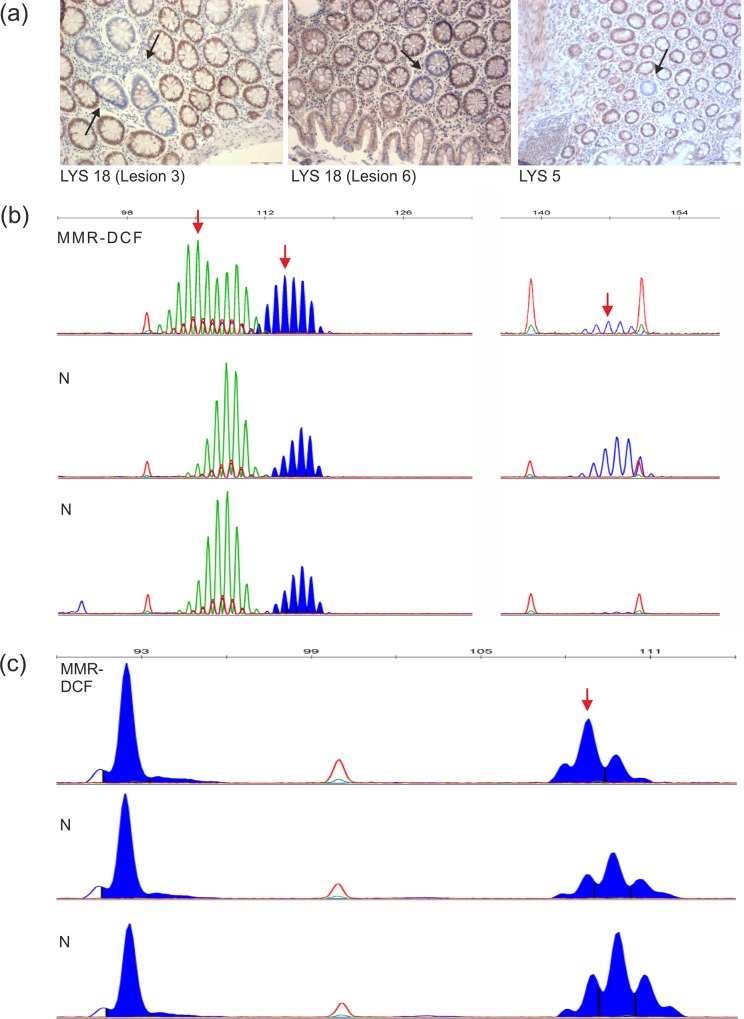
Histology and molecular phenotype of representative MMR-DCF. (A) Staining of three representative MMR-DCF. Objective magnification is 20x for all panels. Representative staining results with antibodies specific for the MMR protein corresponding to the germline mutation of the respective patient. Lesions are denoted by patient ID (LYS = Lynch syndrome patient) and lesion ID. (B) Fragment length analysis of triplex PCR with BAT25, BAT26 and CAT25. Compared to two normal intestinal crypts analysed as a reference (lower panels), all three markers show deletion mutations in the examined MMR-DCF (LYS 21, Lesion 1). Deletion mutations are indicated by red arrows in MMR-DCF (upper panel: BAT25, green peaks, -3; BAT26, filled blue peaks, -2; CAT25, open blue peaks, -1). (C) Fragment length analysis of duplex PCR with *BAX* and *HT001* of patient LYS 19. Depiction of an *HT001* (right sided fragments) mononucleotide shift (-1, red arrow) as an example of a coding microsatellite-bearing target gene mutation. The fragment analysis is directly compared to the results obtained from two normal intestinal crypts of the same patient (lower panels).

In sections from Lynch syndrome patients, immunohistochemistry for MLH1, MSH2 and MSH6 revealed 21 crypt foci lacking the expression of the respective MMR protein (Fig. 1A). The MMR-DCF always showed loss of the MMR protein corresponding to the respective MMR gene germline mutation of the patient. MMR-DCF were identified in the large bowel mucosa of 9 patients and in the small bowel mucosa of 2 patients. Among patients who did not display any MMR-DCF, the maximal length analysed was 28 cm (approximately 1 cm^2^); the minimal length analysed in MMR-DCF-positive patients was 6 cm (0.17 cm^2^) in the small bowel and 8 cm in the colon (0.29 cm^2^).

Overall, the frequency of MMR-DCF in Lynch syndrome mutation carriers was 0.84 foci/cm^2^ (19 foci/22.5 cm^2^) in the colon and 0.83 foci/cm^2^ (2 foci/2.4 cm^2^) in the small bowel. No significant difference of MMR-DCF frequency was observed between MLH1 and MSH2 germline mutation carriers. No MMR-DCF were found in 62 cm (approximately 2.2 cm^2^) mucosa of all 12 patients with sporadic MLH1-hypermethylated MSI-H colorectal cancer patients or in 82 cm (approximately 3 cm^2^) of the control patients without germline mutations of the respective MMR gene.

### Association with clinical parameters

As a next step, a potential correlation of MMR-DCF in the colorectum with tumour localisation, gender and age was addressed. MMR-DCF were significantly more frequent in patients with cancer of the distal colorectum (left colonic flexure or more distal, n = 7) compared to patients with proximal colon cancer (n = 22) (2.38 MMR-DCF per 50 cm [1.8 cm^2^] in the distal colorectum, 95% CI 0.57–4.19, vs. 0.55 MMR-DCF per 50 cm [1.8 cm^2^] in the proximal colon, 95% CI 0.19–0.92, p = 0.008). Patients from both groups, proximal and distal tumour localisation, did not significantly vary concerning parameters such as age and gender (proximal tumour localisation, median age: 44 years, 23% female; distal tumour localisation, median age: 45 years, 14% female).

**Fig 2:**
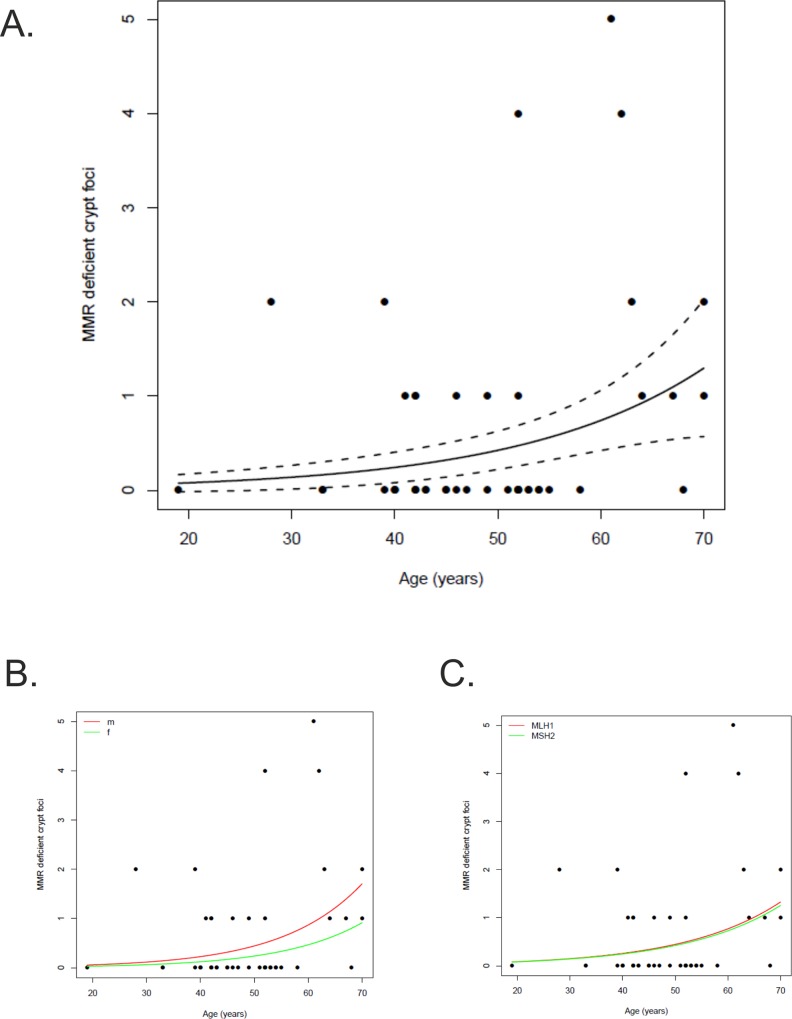
MMR-DCF prevalence and patient age. Association between age (years) of the patient at operation and prevalence of MMR-DCF (A). The association of MMR-DCF prevalence with patient age is depicted with a black line adjusted for a colon length of 15 cm; dots represent patients. Estimated incident rate ratio of colorectal MMR-DCF was 1.75 (95% CI 1.30–2.35) for a change of age at operation of 10 years. The prevalence of MMR-DCF is not significantly associated with patients’ gender (B) or the MMR gene affected by germline mutation (C).

To examine the correlation between prevalence of colorectal MMR-DCF and age or gender, combined analyses encompassing previously published patients [12] were performed. There was no significant association of MMR-DCF prevalence with gender (1.05 MMR-DCF per 50 cm [1.8 cm^2^] in females, 95% CI 0.19–1.92, vs. 0.96 MMR-DCF per 50 cm [1.8 cm^2^] in males, 95% CI 0.57–1.35, p = 0.85). However, the prevalence of colorectal MMR-DCF was significantly related to the age of the patient at the time of the operation (p<0.001). Based on the results of the Poisson regression model, the estimated incident rate ratio of colorectal MMR-DCF was 1.75 (95% CI 1.30–2.35) for a change of age at operation of 10 years. This corresponds to a number of predicted MMR-DCF per 50 cm (1.8 cm^2^) mucosa of 0.59 (95% CI 0.27–0.90) at an age of 40, and 1.03 (95% CI 0.64–1.42) per 50 cm (1.8 cm^2^) at an age of 50, respectively (Fig. 2).

### Morphology

To obtain larger amounts of DNA and more MMR-DCF for morphologic and molecular analysis, paraffin blocks from five of the patients that had revealed MMR-DCF in the first round of staining were resectioned. Using this method, six novel, independent MMR-DCF were identified and included in the molecular analysis. The crypt foci detected in our study displayed a varying size from one to nine crypts. Seven of the now 27 MMR-DCF (4/15 monocryptic, 1/9 oligocryptic and 2/3 polycryptic) presented with dysplastic changes but none showed a polypous or adenomatous appearance. The remaining 20 foci were morphologically undistinguishable from their neighbouring crypts except for their absence of MMR protein expression. Morphological characterisation of MMR-DCF-adjacent mucosal areas after haematoxylin/eosin and immunohistochemistry staining specific for T cell markers including CD4, CD8 and FOXP3 did not reveal signs of an altered immune infiltration in or around any of the MMR-DCF assessed (S4 Fig.).

### Molecular alterations in MMR-DCF

For molecular characterisation of the detected MMR-DCF, we analysed three non-coding and six coding microsatellite markers located in genes proven or suspected to be relevant for tumour formation. Mutation patterns in MMR-DCF were compared to MMR-proficient crypts from Lynch syndrome mutation carriers or MSS colorectal cancer control patients.

The general comparison of microsatellite mutation frequency revealed a significantly higher number of mutation events in MMR-DCF than in MMR-proficient crypts, when all markers were considered: MMR-DCF: 44 out of 186, 23.7%; MMR-proficient crypts from Lynch syndrome mutation carriers: 34 out of 814, 4.2%; MMR-proficient crypts from MSS colorectal cancer patients: 5 out of 173, 2.9%; p<0.001.

The mutation frequency observed in MMR-DCF was highest for the three non-coding microsatellite markers BAT25, BAT26, and CAT25, which showed a mutant pattern in 36%, 57%, and 60% of the analysed MMR-DCF, respectively (Fig. 1B). No significant difference was observed between the three markers.

Among coding microsatellites, the observed mutation frequency ranged between 0% and 33% for the six analysed markers located in the coding regions of the genes *AIM2*, *ACVR2*, *BAX*, *HT001*, *TAF1B* and *TGFBR2* (Fig. 1C). Mutations were detected in 5 out of 6 markers tested, but were absent in *TGFBR2*. Mutation frequency was highest for the *HT001* microsatellite (33%) followed by *AIM2* (17%) and *BAX* (10%). For these three markers the difference of mutation prevalence between MMR-DCF and MMR-proficient crypts was significant: *HT001*: 7/21 (33%) vs. 0/72 (0%), p<0.0001; *AIM2*: 4/24 (17%) vs. 2/85 (2%), p = 0.0206; *BAX*: 2/21 (10%) vs. 0/75 (0%), p = 0.0461. Mutation frequency of non-coding and coding microsatellites is displayed in Table 1.

**Table 1:** Overview of mutation frequencies of all markers tested. Absolute numbers of crypts examined are followed by mutation percentages. MMR-proficient crypts from Lynch syndrome patients and MSS colorectal cancer patients were evaluated as controls. P-values were calculated concerning results of MMR-DCF versus Lynch syndrome crypts.

	MMR-DCF	MMR-proficient crypts		p-value
		LS mucosa	MSS CRC mucosa	
**BAT25**	8/22 (36%)	12/135 (9%)	3/26 (12%)	0.0018
**BAT26**	12/21 (57%)	9/121 (7%)	1/25 (4%)	<0.0001
**CAT25**	9/15 (60%)	6/85 (7%)	0/14 (0%)	<0.0001
**AIM2**	4/24 (17%)	2/85 (2%)	1/18 (6%)	0.0206
**ACVR2**	1/21 (5%)	0/72 (0%)	0/16 (0%)	0.2258
**BAX**	2/21 (10%)	0/75 (0%)	0/17 (0%)	0.0461
**HT001**	7/21 (33%)	0/72 (0%)	0/14 (0%)	<0.0001
**TAF1B**	1/20 (5%)	2/90 (2%)	0/24 (0%)	0.4557
**TGFBR2**	0/21 (0%)	3/79 (4%)	0/19 (0%)	1.000

**Fig 3:**
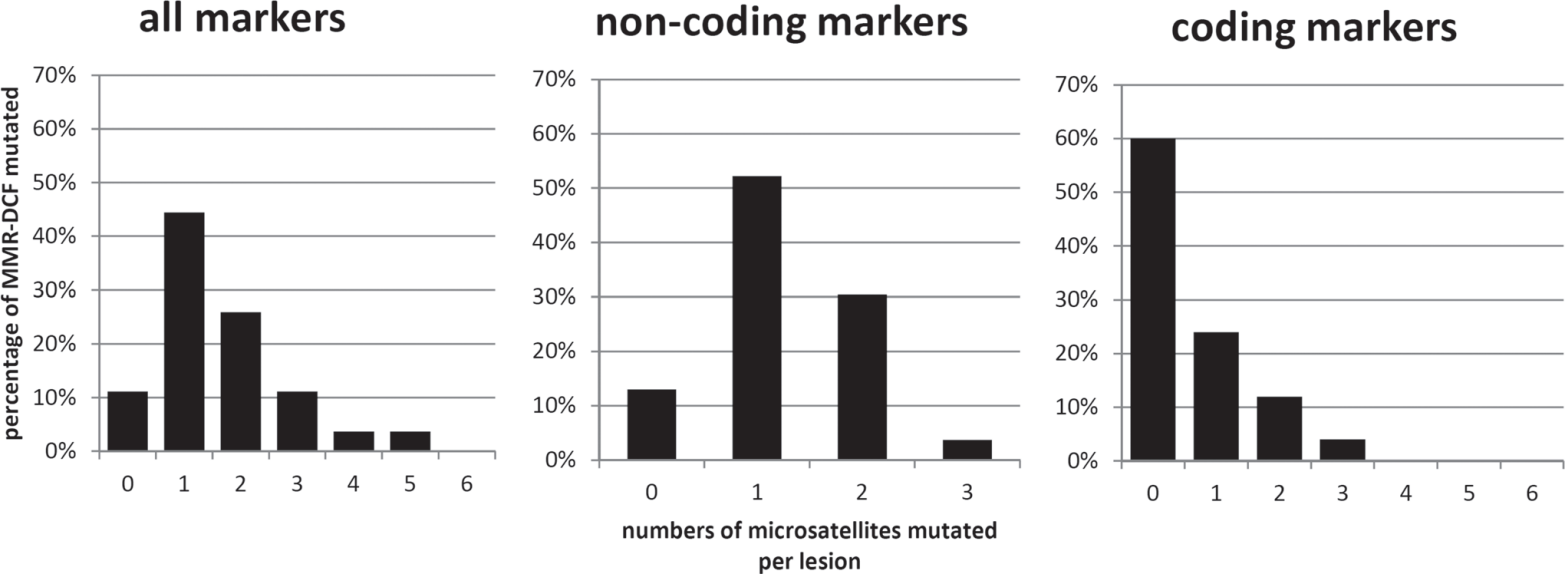
Distribution of mutation patterns within MMR-DCF. Numbers of microsatellites mutated per MMR-DCF, separately shown for all markers (left panel), non-coding markers (middle: BAT25, BAT26, and CAT25), and coding markers (right panel: *AIM2*, *ACVR2*, *BAX*, *HT001*, *TAF1B* and *TGFBR2*) are separately shown. The y-axis represents the percentage of MMR-DCF mutated, whereas the x-axis displays the numbers of microsatellites affected per lesion.

From 23 crypt foci evaluable for MSI at non-coding markers, 20 (87%) presented with mutations affecting at least one of the markers BAT25, BAT26, or CAT25. MSI affecting coding markers was detected in 10 out of 25 (40%) of MMR-DCF. The distribution of coding and non-coding marker mutation frequency per individual crypt is illustrated in Fig. 3. MMR-DCF consisting of more than one crypt tended to show a higher frequency of mutations than single-crypt MMR-DCF, though statistical significance was lacking (23/100 vs. 21/130, p = 0.24, Fisher’s exact test).

### Regional diversity of MMR-DCF

To examine the mutational events taking place during the clonal expansion of MMR-deficient crypts, we examined regional diversity of mutations in MMR-DCF by separately analysing six regions of one MLH1-negative extended crypt focus from patient LS 4 (extended set). Mutations were only detected in *HT001* and *AIM2* from all the coding markers, and *HT001* presented with the most heterogeneous mutation pattern. For the non-coding markers, there were mutations displayed in almost every region of the crypt focus, also differing in the mutation pattern (S3 Fig.).

### Comparison of the mutation frequency in MMR-DCF and MSI-H colorectal cancer

**Fig 4:**
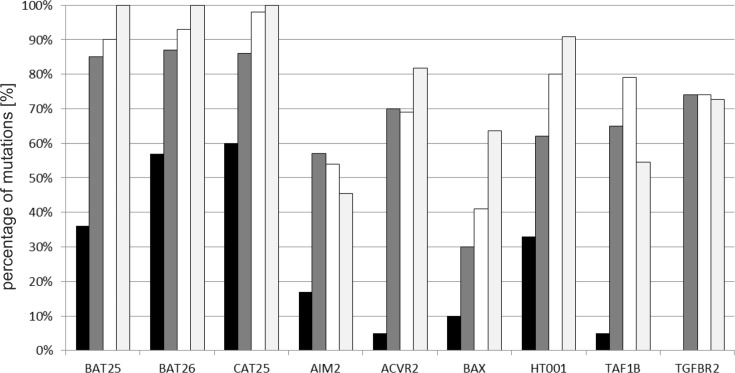
Mutation frequency of microsatellite markers. Black bars represent the observed mutation frequency of the respective microsatellites in MMR-DCF. Mutation frequency reported in the literature for MSI-H colorectal adenoma and carcinoma are displayed by dark grey (adenoma) and white (carcinoma) bars, respectively. Data for adenomas and carcinomas were retrieved from www.seltarbase.org. Light grey bars indicate mutation frequency in corresponding carcinomas from the same patients from whom the MMR-DCF were obtained.

In order to compare the mutation frequency of selected microsatellite markers between MMR-DCF and clinically manifest MSI-H adenomas and carcinomas, data for MSI-H colorectal adenoma and cancer were retrieved from SelTarbase [15]. In addition, cancer specimens obtained from the study patients were analysed. As shown in Fig. 4, the mutation prevalence of the three non-coding microsatellite markers BAT25, BAT26 and CAT25 in MMR-DCF followed the same tendency as observed in MSI-H colorectal cancer. CAT25 presented with the highest mutation rate, followed by BAT26 and BAT25 in both groups. Of the six coding microsatellite markers, *HT001* presented with the highest mutation frequency in both groups. In contrast, the most pronounced discrepancies were observed for *TAF1B* and *TGFBR2* (Fig. 4).

## Discussion

In the present study, we focused on molecular alterations occurring in MMR-DCF, and their relation to clinical or histopathological parameters. Our observations confirm the previously described prevalence of approximately one MMR-DCF per 1 cm^2^ of mucosa [12].

For the first time, patients with sporadic MSI-H colorectal cancer with *MLH1* promoter hypermethylation were analysed for the presence of MMR-DCF. No MMR-DCF were observed in these patients, including the directly tumour-adjacent mucosa, which could be assessed from all patients. The lack of MMR-DCF in patients with sporadic MSI-H colorectal cancer may result from a distinct pathway of tumour development that, in contrast to abrupt loss of MLH1 in Lynch syndrome-associated lesions, encompasses a gradual decrease of MLH1 protein expression due to increasing epigenetic changes [23,24]. Our observations suggest that the occurrence of MMR-DCF is restricted to Lynch syndrome mutation carriers and closely associated with the presence of MMR gene germline mutations. It is therefore expected that patients with sporadic MSI-H colorectal cancers that develop cancer as a consequence of two somatic MMR gene mutations [25–28] will not harbour MMR-deficient crypt foci in the colon mucosa, similar to our observation in patients with *MLH1*-hypermethylated cancers. However, so far no data are available to confirm this assumption. A strong correlation was observed between patient’s age at operation and the prevalence of MMR-DCF (p<0.001). This finding indicates an increasing frequency of MMR-DCF in the mucosa from Lynch syndrome mutation carriers with increasing patients’ age. It is not clear, though, whether this results from accumulation and persistence of all MMR-DCF formed during life, or whether it reflects a higher rate of lesion formation than lesion elimination. An additional factor that might contribute to the accumulation of MMR-DCF in elderly people could be an increasing susceptibility of existing cells towards developing a mutation due to genomic instability [29]. This question cannot be resolved on the basis of the present study, because the cross-sectional study design does not allow any conclusions on the turnover of MMR-DCF.

In Lynch syndrome patients, colorectal cancer predominantly develops in the proximal colon [9]. However, a higher frequency of MMR-DCF was observed in Lynch syndrome patients with distal colorectal cancer localisation. This observation may suggest that in Lynch syndrome patients with distal colorectal carcinoma, unidentified factors affecting the carcinogenesis may also promote the development of MMR-DCF. A potential association between the number of MMR-DCF and the exposure towards DNA-damaging substances is in fact suggested by a significant increase of mismatch repair-deficient crypts upon temozolomide treatment in a mouse model of Lynch syndrome [30]. The localisation of colorectal cancer to the proximal or distal colorectum in our study was not related to patients’ age and gender, excluding these parameters as potential influencing factors.

No significant difference with regard to MMR-DCF prevalence was observed between men and women. Accordingly, we did not observe any evidence that a difference of MMR-DCF incidence may be responsible for the higher estimated colorectal cancer risk of male compared to female Lynch syndrome mutation carriers [31,32]. Similarly, MMR-DCF prevalence was identical between MLH1 and MSH2 mutation carriers, suggesting that the gene affected by germline mutation is not a predictor of MMR-DCF formation.

Immune surveillance may contribute to the recognition of MMR-deficient cells by cytotoxic T cells, potentially following the generation of MMR deficiency-induced frameshift peptide antigens [33,34]. However, morphological characterisation of MMR-DCF-adjacent mucosal areas after haematoxylin/eosin and immunohistochemistry staining specific for T cell markers did not reveal significant alterations of immune infiltration in or around any of the MMR-DCF assessed (S4 Fig.). These results indicate that increased immune cell infiltration was not a general phenomenon in the vicinity of MMR-DCF. However, the lack of increased immune infiltration in paraffin-embedded tissue sections does not preclude a functionally relevant, specific local immune activation. One also has to take into account that immunologic elimination of crypt foci may take place in a very short time compared to the persistence of MMR-DCF and thereby escape visual detection. In fact, an enhanced density of mucosal lymphocytes has recently been described in one MMR-deficient crypt focus [35].

The possibility of examining DNA from individual MMR-deficient crypts opens a window towards assessing the initial molecular changes occurring after loss of MMR function. Mutation analyses in frame of our previous study had already revealed mutations affecting large, non-coding microsatellite markers, which are highly prone to insertion/deletion mutations in the context of MMR deficiency, in the majority of MMR-DCF [12]. In the present study, the molecular examination of MMR-DCF was largely extended. For the first time, single-crypt MMR-DCF were examined for molecular alterations. Our results demonstrate mutations of the three non-coding microsatellite markers BAT25, BAT26, and CAT25 in 40–60% of the MMR-DCF. In total, 87% of all MMR-DCFs showed a mutation in at least one of the MSI markers, indicating that the loss of MMR protein expression has immediate functional consequences, already in small and mono-cryptic MMR-DCF. Moreover, they underline the suitability of the markers BAT25, BAT26, and CAT25 for the detection of the earliest stages of MMR deficiency in the colorectum.

For the first time, mutations of several coding microsatellites could be assessed simultaneously in MMR-DCF. Five of the six coding microsatellites assessed in this study displayed mutations in MMR-DCF (*AIM2*, *ACVR2*, *BAX*, *HT001*, and *TAF1B)*. The highest mutation frequency (33%) in the present study was detected for *HT001*, followed by *AIM2* and *BAX*. The observation of *HT001* mutations in one third of the analysed MMR-DCF may be compatible with a potential relevance of the respective gene. A potential tumour-suppressive function of *HT001* is further supported by the high mutation frequency in MSI-H colorectal and endometrial cancer [18]. In general, the comparison of coding microsatellite mutation frequencies follow a similar trend in MMR-DCF compared to corresponding MSI-H cancers from the same patients and to published data for colorectal adenomas and carcinomas [15]. The observed regional heterogeneity in one larger crypt focus (S3 Fig.) suggests that MMR-DCF represent a novel model lesion for studying molecular evolution in Lynch syndrome [36,37], and early events associated with initiation and progression of solid neoplasia in general.

The comparatively high prevalence of *HT001* and *AIM2* mutations in MMR-DCF is also interesting with regard to frameshift peptide vaccination approaches, which are currently evaluated in MSI-H colorectal cancer patients. The Micoryx vaccine (clinicaltrials.gov, NCT01461148) encompasses antigens, which result from mutations of *HT001* and *AIM2*. Consequently, frameshift peptide vaccination may represent a promising approach for tumour prevention in Lynch syndrome mutation carriers.

Interestingly, no mutations of the *TGFBR2* coding microsatellite were observed in the present study, which is in contrast to our previous study [12], where *TGFBR2* mutations were detected in larger polycryptic foci. This discrepancy suggests that the occurrence of *TGFBR2* mutations is restricted to large MMR-DCF that already show morphological alterations [12]. Hence, *TGFBR2* mutations represent promising candidate alterations that might contribute to the progression of MMR-DCF towards malignant transformation of MMR-DCF. Further studies are needed to validate this hypothesis.

To exclude technical reasons as factors underlying the observed alterations in MMR-DCF (e.g. related to small amounts of template DNA or formalin fixation), we compared the mutation frequency of microsatellite markers in MMR-DCF and MMR-proficient mucosa from Lynch syndrome mutation carriers or MSS colorectal cancer patients. The comparison revealed a low background rate of mutations. The detection of significantly higher rates of mutations in MMR-DCF clearly argues in favour of MMR deficiency as a biological reason underlying the detected mutations. Moreover, we were not able to confirm previous reports about a significantly higher prevalence of microsatellite mutations in normal mucosa from Lynch syndrome mutation carriers compared to MSS colorectal cancer control patients [38].

## Conclusions

In summary, we here demonstrate for the first time that microsatellite instability is detectable in the vast majority of MMR-DCF, including monocryptic lesions. Clinically, the occurrence of MMR-DCF in Lynch syndrome mutation carriers is significantly correlated to age, suggesting ongoing accumulation of MMR-DCF. Coding microsatellite mutations were observed in mono- and poly-cryptic MMR-DCF, most frequently affecting *HT001*, *AIM2* and *BAX*. Mutations of the *TGFBR2* gene appear to be restricted to polycryptic MMR-DCF, potentially suggesting that they may favour progression or malignant transformation.

## Supporting Information

S1 Fig Immunohistochemical stains of MMR-DCF. (A) Immunohistochemical stains of all initial MMR-DCF. Overview of staining results with antibodies specific for the MMR protein corresponding to the germline mutation of the respective patient. Lesions are denoted by patient ID and lesion ID. All pictures were taken with a 20x magnification except for LYS 18 (Lesion 4) where a 10x magnification was used. (B) Immunohistochemical stains of six additional MMR-DCF. Lesions are denoted by patient ID and lesion ID and represent resectioning results of the respective patients. For all pictures a 20x magnification was used.
(TIF)

S2 Fig Combined overview of all 34 Lynch syndrome patients. This diagram presents all 34 Lynch syndrome patients with patient ID,age at operation, tumour localisation and operation performed (SC = subtotal colectomy, RH = right hemicolectomy, LH = left hemicolectomy, AR = anterior rectum resection, SR = sigma resection and TR = total rectum resection). Green bars correspond to the primary x-axis displaying total numbers of MMR-DCF (first eleven patients). Adjacent light blue (men) and light pink (women) bars indicate the measured mucosal length of the respective patient.
(TIF)

S3 Fig Regional diversity of MMR-DCF. (A) Regional diversity in non-coding markers BAT25, BAT26 and CAT25. The observed mutations varied widely between the six regions examined in this PCR approach for BAT25 (green), BAT26 (blue, filled) and CAT25 (blue). na = not available. BAT25: Region 1 (-3), Region 2 (-3), Region 3 (-3), Region 4 (wt), Region 5 (-2), Region 6 (-3). BAT26: Region 1 (-4), Region 2 (-6), Region 3 (-3), Region 4 (-3), Region 5 (-3), Region 6 (-4). CAT25: Region 1 (+3), Region 2 (+1), Region 3 (+2), Region 4 (na), Region 5 (wt), Region 6 (-1). (B) Regional diversity in the coding marker *HT001*. Regional diversity in the coding marker *HT001* for one extensive crypt focus which was divided into six separately analysed regions. The observed shift mutations were evaluated as follows: Region 1 (-1), Region 2 (-1), Region 3(-2), Region 4 (-1), Region 5 (-1) and Region 6 (-1). (C) Microscopic picture of lesion 3 (patient LS 4). Objective magnification is 10x. The extensive MMR-DCF was divided into six separately analysed regions from the left (region 1) to the right (region 6) side.
(TIF)

S4 Fig Representative example of mucosal immune cells surrounding MMR-DCF. To evaluate the local immune infiltration, serial sections were stained with immune cell markers (CD4, CD8 and FOXP3). No difference in immune cell infiltration was observed in MMR-DCF and surrounding regions. Immune cell infiltration of MMR-DCF (black arrows) was compared to two control regions in the vicinity (2 mm and 10 mm distant) of the respective lesion. All images are shown at 20x magnification.
(TIF)
