# Supplementary figures and images for: Mismatch Repair-Deficient Crypt Foci in Lynch Syndrome – Molecular Alterations and Association with Clinical Parameters
Source: PLoS One. 2015 Mar 27;10(3):e0121980. doi: 10.1371/journal.pone.0121980 (PMC4376900; doi:10.1371/journal.pone.0121980)

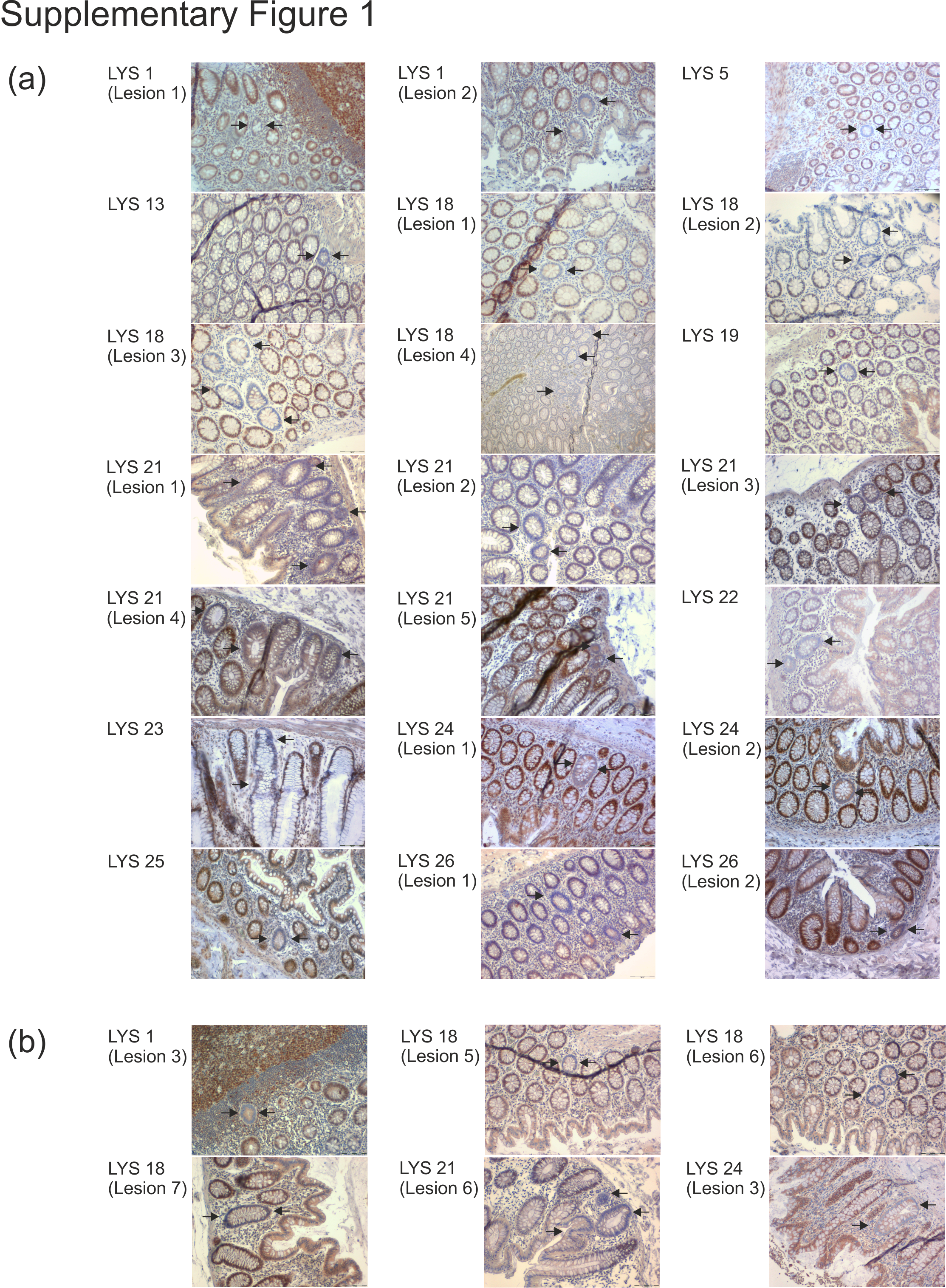

Supplement: S1 Fig — (A) Immunohistochemical stains of all initial MMR-DCF. Overview of staining results with antibodies specific for the MMR protein corresponding to the germline mutation of the respective patient. Lesions are denoted by patient ID and lesion ID. All pictures were taken with a 20x magnification except for LYS 18 (Lesion 4) where a 10x magnification was used. (B) Immunohistochemical stains of six additional MMR-DCF. Lesions are denoted by patient ID and lesion ID and represent resectioning results of the respective patients. For all pictures a 20x magnification was used. (TIF) [file pone.0121980.s001.tif]

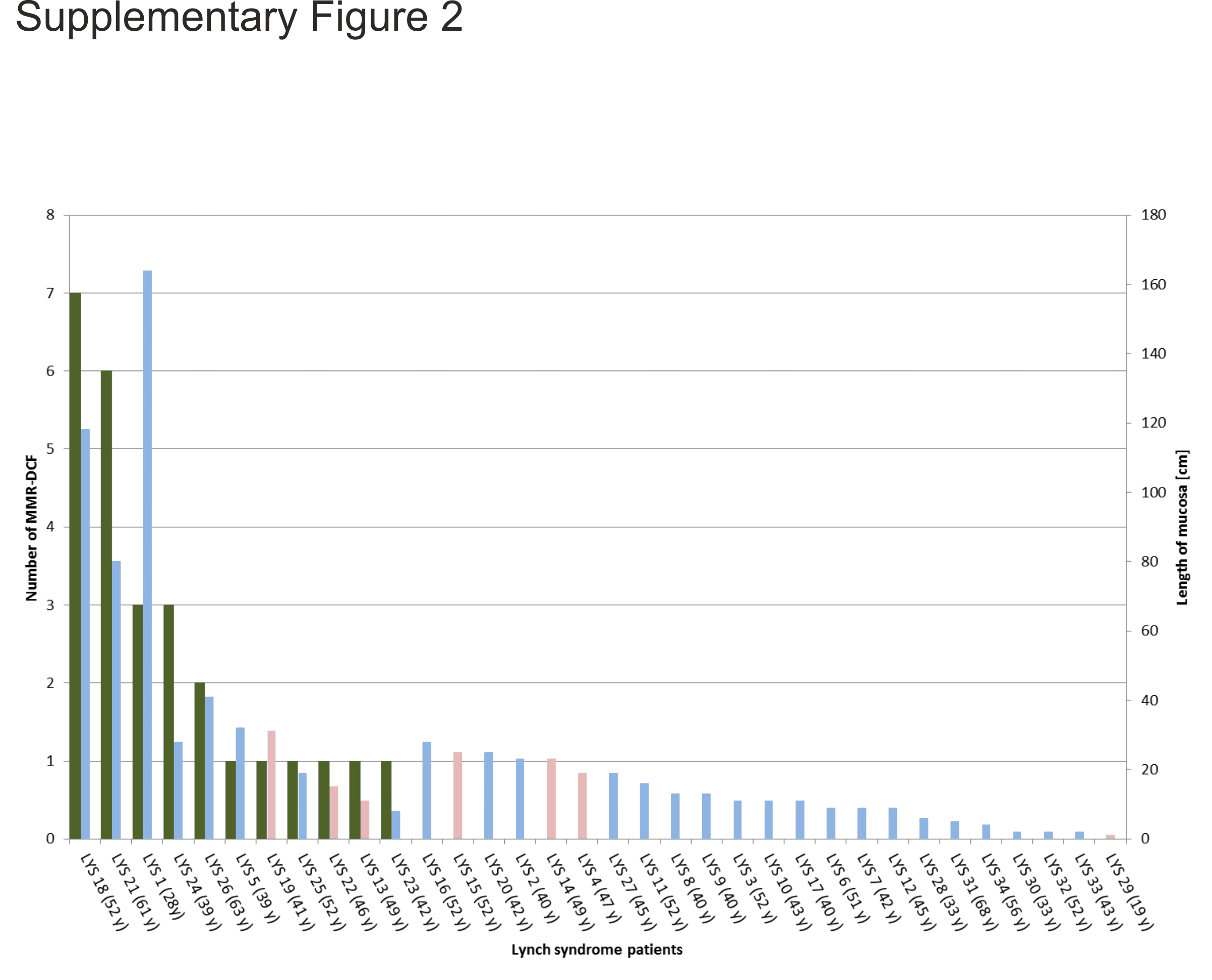

Supplement: S2 Fig — This diagram presents all 34 Lynch syndrome patients with patient ID,age at operation, tumour localisation and operation performed (SC = subtotal colectomy, RH = right hemicolectomy, LH = left hemicolectomy, AR = anterior rectum resection, SR = sigma resection and TR = total rectum resection). Green bars correspond to the primary x-axis displaying total numbers of MMR-DCF (first eleven patients). Adjacent light blue (men) and light pink (women) bars indicate the measured mucosal length of the respective patient. (TIF) [file pone.0121980.s002.tif]

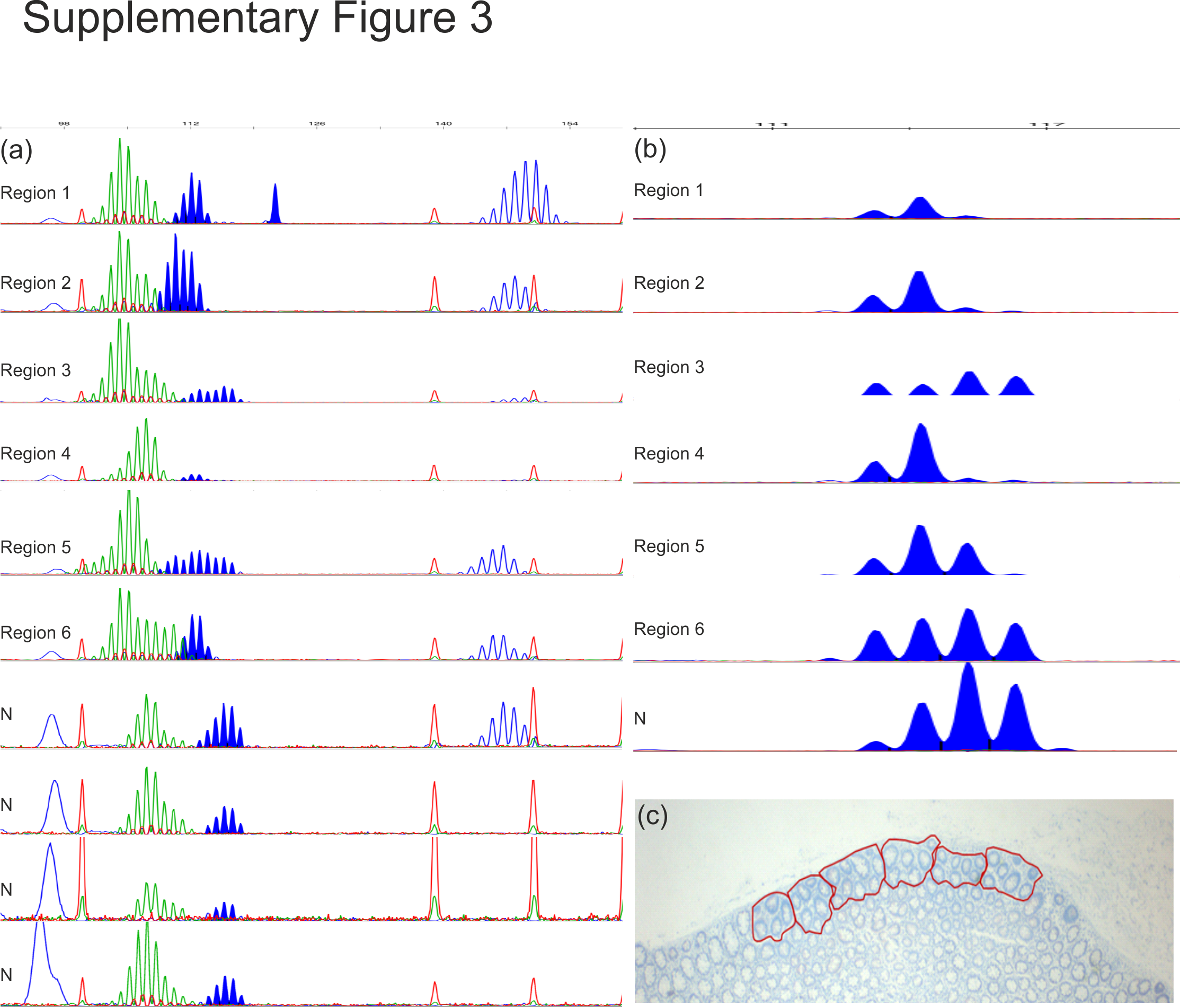

Supplement: S3 Fig — (A) Regional diversity in non-coding markers BAT25, BAT26 and CAT25. The observed mutations varied widely between the six regions examined in this PCR approach for BAT25 (green), BAT26 (blue, filled) and CAT25 (blue). na = not available. BAT25: Region 1 (-3), Region 2 (-3), Region 3 (-3), Region 4 (wt), Region 5 (-2), Region 6 (-3). BAT26: Region 1 (-4), Region 2 (-6), Region 3 (-3), Region 4 (-3), Region 5 (-3), Region 6 (-4). CAT25: Region 1 (+3), Region 2 (+1), Region 3 (+2), Region 4 (na), Region 5 (wt), Region 6 (-1). (B) Regional diversity in the coding marker HT001. Regional diversity in the coding marker HT001 for one extensive crypt focus which was divided into six separately analysed regions. The observed shift mutations were evaluated as follows: Region 1 (-1), Region 2 (-1), Region 3(-2), Region 4 (-1), Region 5 (-1) and Region 6 (-1). (C) Microscopic picture of lesion 3 (patient LS 4). Objective magnification is 10x. The extensive MMR-DCF was divided into six separately analysed regions from the left (region 1) to the right (region 6) side. (TIF) [file pone.0121980.s003.tif]

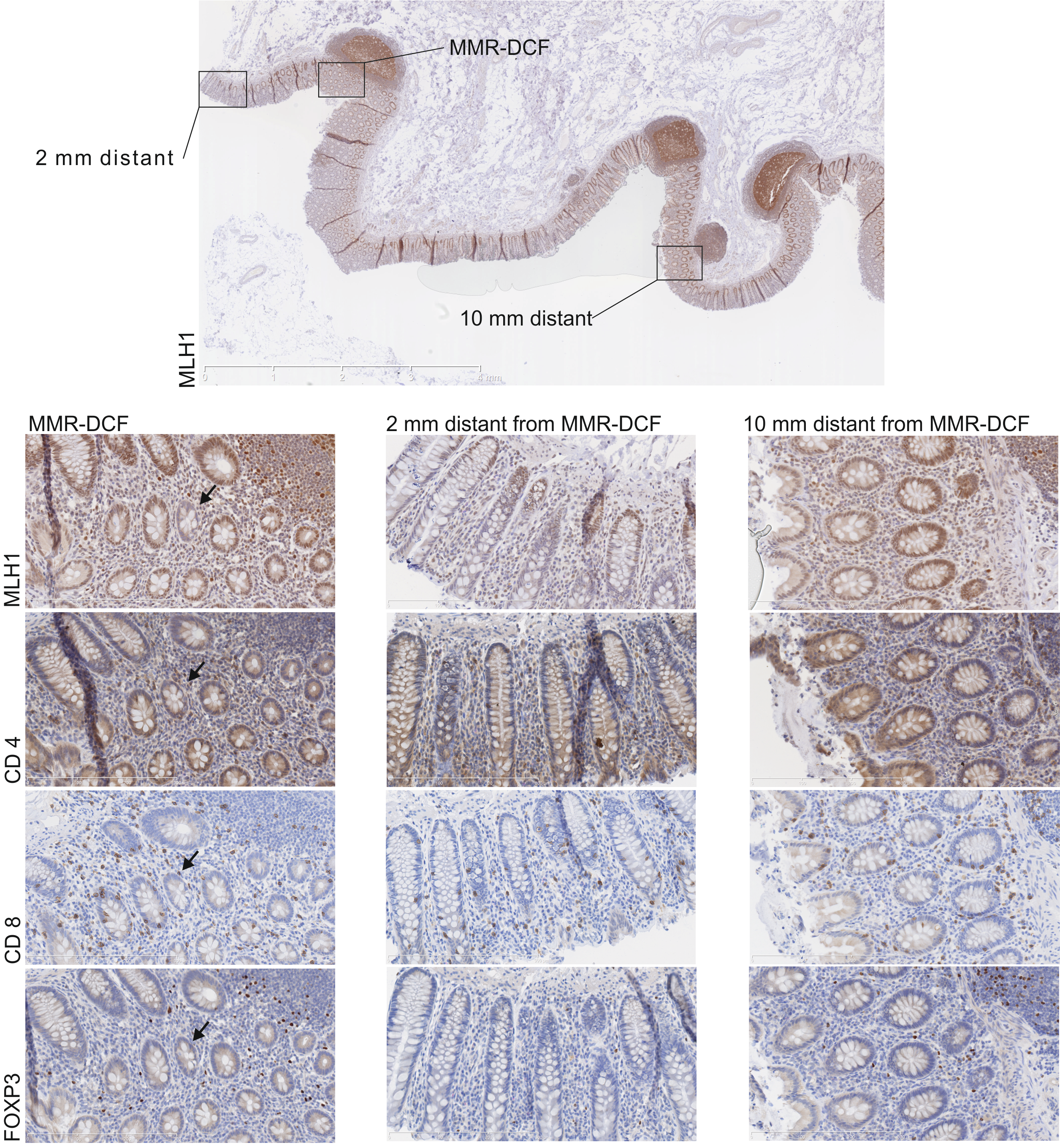

Supplement: S4 Fig — To evaluate the local immune infiltration, serial sections were stained with immune cell markers (CD4, CD8 and FOXP3). No difference in immune cell infiltration was observed in MMR-DCF and surrounding regions. Immune cell infiltration of MMR-DCF (black arrows) was compared to two control regions in the vicinity (2 mm and 10 mm distant) of the respective lesion. All images are shown at 20x magnification. (TIF) [file pone.0121980.s004.tif]
